# Computational Structure Prediction for Antibody-Antigen Complexes From Hydrogen-Deuterium Exchange Mass Spectrometry: Challenges and Outlook

**DOI:** 10.3389/fimmu.2022.859964

**Published:** 2022-05-26

**Authors:** Minh H. Tran, Clara T. Schoeder, Kevin L. Schey, Jens Meiler

**Affiliations:** ^1^ Chemical and Physical Biology Program, Vanderbilt University, Nashville, TN, United States; ^2^ Center of Structural Biology, Vanderbilt University, Nashville, TN, United States; ^3^ Mass Spectrometry Research Center, Department of Biochemistry, Vanderbilt University, Nashville, TN, United States; ^4^ Department of Chemistry, Vanderbilt University, Nashville, TN, United States; ^5^ Institute for Drug Discovery, University Leipzig Medical School, Leipzig, Germany

**Keywords:** hydrogen-deuterium exchange mass spectrometry (HDX-MS), antibody-antigen interaction, epitope-paratope identification, protein-protein docking, structure modeling, integrative structural biology

## Abstract

Although computational structure prediction has had great successes in recent years, it regularly fails to predict the interactions of large protein complexes with residue-level accuracy, or even the correct orientation of the protein partners. The performance of computational docking can be notably enhanced by incorporating experimental data from structural biology techniques. A rapid method to probe protein-protein interactions is hydrogen-deuterium exchange mass spectrometry (HDX-MS). HDX-MS has been increasingly used for epitope-mapping of antibodies (Abs) to their respective antigens (Ags) in the past few years. In this paper, we review the current state of HDX-MS in studying protein interactions, specifically Ab-Ag interactions, and how it has been used to inform computational structure prediction calculations. Particularly, we address the limitations of HDX-MS in epitope mapping and techniques and protocols applied to overcome these barriers. Furthermore, we explore computational methods that leverage HDX-MS to aid structure prediction, including the computational simulation of HDX-MS data and the combination of HDX-MS and protein docking. We point out challenges in interpreting and incorporating HDX-MS data into Ab-Ag complex docking and highlight the opportunities they provide to build towards a more optimized hybrid method, allowing for more reliable, high throughput epitope identification.

**Graphical Abstract:**
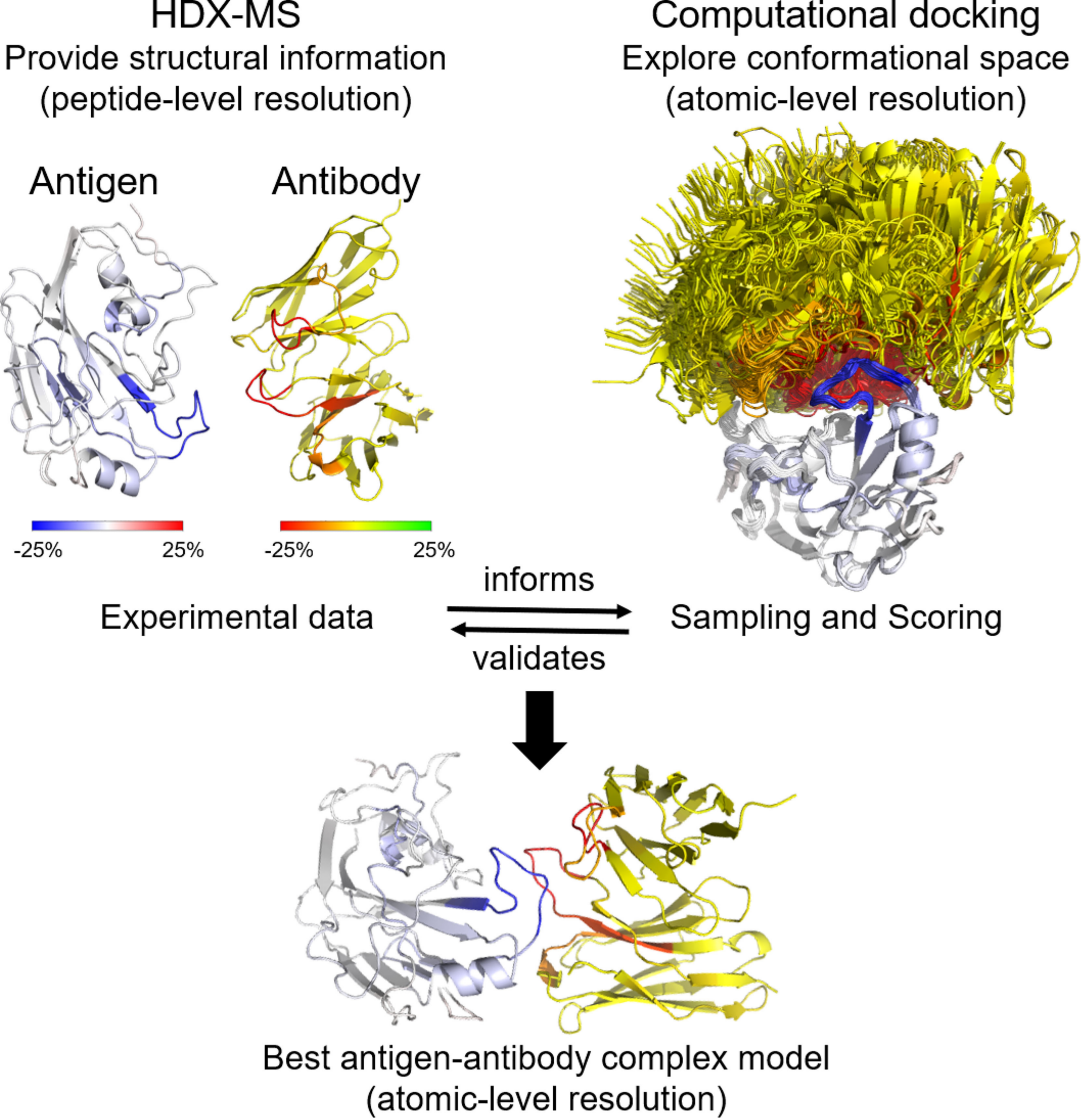


## Introduction

The structural characterization of Ab-Ag interactions has become increasingly important throughout the last decade. The affinity and specificity of monoclonal Abs make them very effective medical therapies and the top growing drug classes in the past few years (1–5). As a result, there is a rising need for enhanced bioanalytical methods to characterize the structural basis of Ab-Ag recognition. Correct identification of critical residues for Ab engineering informs Ab engineering to improve its affinity and specificity or binding breadth for therapeutic applications and Ag design to produce prophylactic or therapeutic vaccines for disease prevention (6–9).

Methods that can rapidly map epitopes play a crucial role in guiding early-stage Ab development and provide critical information for early decision making and Ab selection. Nevertheless, this task remains challenging (10). Existing epitope mapping methods, such as electron microscopy (EM), X-ray crystallography, bio-layer interferometry, and site-directed mutagenesis can be expensive, have limited-throughput, and still might not capture the binding interface of Ab-Ag complexes (7). Some of these methods only recognize linear epitopes, while 80-90% of Ab-Ag interactions have at least one conformational epitope (11, 12).

Computationally, Ab-Ag complexes can be predicted using protein-protein docking. The challenges that come with accurate prediction of Ab-Ag complexes are two-fold: the sampling problem where too many potential binding interfaces need to be explored, and the scoring problem where the native model must be accurately ranked and chosen from a big pool of generated outputs. The integration of experimentally derived information at the sampling and scoring stage of docking has been shown to yield more accurate models by focusing the potential sampling conformations and distinguishing correct predicted models from the rest (13, 14).

HDX-MS is a powerful technique used increasingly to study protein-protein interactions. It can be combined with computational protein modeling protocols to produce high-resolution near-native models. In this review, we will discuss how HDX-MS has been applied to map Ab-Ag interactions and delineate the limitations of the HDX-MS experiment and data interpretation. We will emphasize the limitations that computation has been unable to resolve fully, including the simulation of HDX-MS data from protein atomic structure as well as the lack of quantitative restraints, inaccurate data interpretation, and non-optimized scoring functions for the use of HDX-MS data during protein-protein docking. Our specific interest lies in employing HDX-guided computational structure prediction to model Ab-Ag complexes.

## HDX-MS Overview: Fundamentals, Applications, and Advantages as an Epitope Mapping Tool

HDX-MS measures the change in mass of a protein after its exposure to deuterated solvent, resulting from the isotopic exchange between protein backbone amide hydrogens and deuterium (15–18). To measure deuterium exchange for specific peptides, the protein is labeled in the presence of D_2_O and fragmented by proteolysis. The backbone amide hydrogens free of hydrogen bond and/or exposed to the solvent typically exchange more rapidly compared to those participating in stable hydrogen bond or shielded from the solvent. The rate and location of deuterium incorporation provide fundamental biophysical information about protein folding pathways, localized structural conformation of proteins, and protein interactions.

While standard biophysical methods such as X-ray crystallography provide static models of a protein, HDX-MS offers valuable information about protein dynamics in addition to its structure. HDX-MS is applicable to numerous study areas: structural-function relationships, membrane proteins, protein folding and refolding, impacts of post-translational modifications, sequence change, or denaturation, and protein binding analysis (19, 20). More specifically, HDX-MS has been employed to examine protein conformations, to study protein intrinsically disordered regions (21), to study conformational dynamics after modification (i.e., compare the dynamic change of H7 HA0 trimer and cleaved HA trimer) (22), and to monitor protein folding pathways and quality control [i.e., biopharmaceutical comparability studies detected subtle conformational differences between protein samples (20) or detected potential aggregation interfaces (23)]. HDX-MS is also performed to locate protein-ligand binding sites as well as protein-protein interactions (18, 24).

HDX-MS has been increasingly applied as an epitope mapping tool due to its many advantages (25, 26). The biggest advantage of HDX-MS is that it is fast and cost-effective, thus achieving a relatively high throughput. With the development of HDX-MS data processing software and a routine workflow, an epitope mapping study can be completed within one week in ideal situations (27). Its rapid turnaround time can be beneficial in urgent public health situations when Ab profiling is necessary for Ab therapeutic development (28). In addition, HDX-MS requires minimal amounts of protein material (μl of the sample at low μM concentrations), which renders it applicable for the characterization of large panels of Ab-Ag complexes (19, 29). Furthermore, HDX-MS is unrestricted by the size of the proteins because they are cleaved into peptic peptides prior to deuterium uptake analysis. In fact, HDX-MS is one of few technologies that can be employed to study the local conformational dynamics of hemagglutinin, a major surface protein of influenza viruses in solution, because these complexes are very large (25). Generally, *de novo* structure determination by nuclear magnetic resonance (NMR) is challenging for proteins larger than 50 kDa. Hence, Ab-Ag complexes are not good targets for traditional NMR studies because most of them easily exceed 50kDa (30). All these advantages make HDX-MS a very potent and rapid method to study Ab-Ag complexes, as well as proteins that are unamenable to other structural methods due to their intrinsic nature.

## HDX-MS Experimental Approach and Data Processing for Ab-Ag Structure Prediction

**Figure 1:**
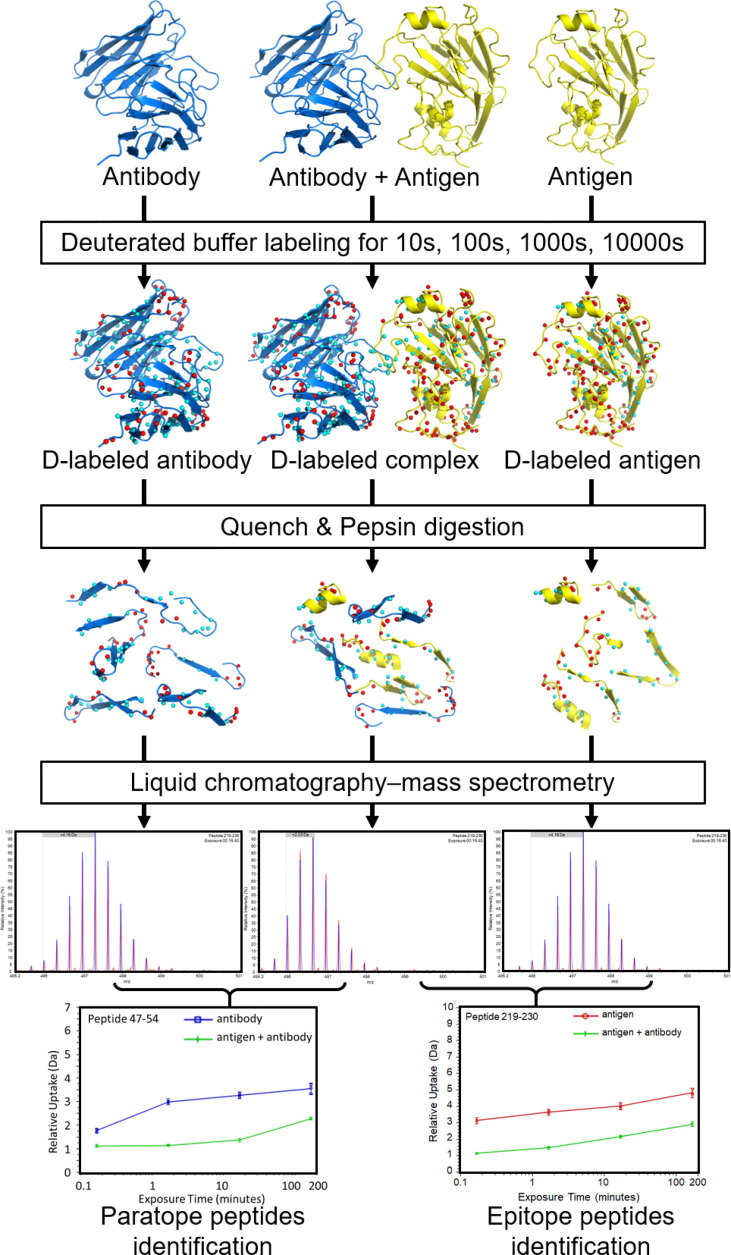
Overview of a typical workflow for an epitope/paratope mapping HDX-MS experiment. Separately, the Ag, Ab-Ag complex, and Ab are labeled in D_2_O and incubated for varying lengths of time. The reactions are then quenched at low pH and low temperature. The protein samples are digested (typically with pepsin) to generate peptide fragments. Peptide fragments from each sample are analyzed using LC-MS to identify mass differences at various time points. The D uptake altered by binding enables identification of putative paratope and epitope peptides. Figure is adapted from (20).

HDX-MS has been used successfully in the past to map Ab epitopes to Ag surfaces (e.g., 14, 20, 22, 28, 31–36). A typical HDX-MS epitope mapping experiment (**Figure 1**) is performed on the Ag alone as a reference and then in complex with the Ab. The protein samples are labeled in D_2_O buffer under equilibrium conditions for each of several different time points. The binding of an Ab reduces the solvent exposure of Ag residues residing in the binding interface. This lowers the level of deuterium incorporation into peptic fragments containing the affected residues. Therefore, comparing HDX-MS profiles between Ab-free and Ab-bound states enables the identification of potential epitope peptides. Optionally, another set of labeling runs on the Ab by itself is added for paratope mapping (**Figure 1**).

After labeling, quenching is performed immediately by a drop in pH and temperature to minimize back exchange (37). Denaturants and reducing agents (i.e., tris(2-carboxyethyl)phosphine (TCEP)) are often added to the quench solution to reduce disulfide bonds in Abs because they are highly protease-resistant and will complicate MS data interpretation if left intact (19, 38). After pepsin digest (typically on-column), proteolytic peptides are desalted, separated on an ultra-high-performance liquid chromatography (LC) column, and ionized by electrospray ionization (ESI). The peptides’ masses are measured. By comparing the mass in the complex sample and in free sample for each peptide, its deuterium incorporation is computed, revealing potential peptides participating in the Ab-Ag interface (19).

Overall, the output of a conventional HDX-MS experiment is the level of deuterium uptake of each individual peptide at each labeling time point, accumulated by amide groups constituting that peptide. In most epitope mapping studies, the exchange rate of each peptide is averaged over all its amide groups, compared between the complex state and free state, and mapped onto the Ag/Ab structure if available (14, 20, 22, 31–35). These peptide-resolution HDX-MS data preclude quantitative analysis and identification of residue interactions between Ab and Ag (39).

HDX-MS provides the most comprehensive understanding of Ab-Ag interactions when combined with other structural techniques such as chemical-crosslinking with MS (XL-MS), cryo-electron microscopy (cryo-EM), or X-ray crystallography. XL-MS delivers information about the proximity of nearby protein sites and the distances between specific residues due to the identified inter-molecular cross-links. When combined with HDX-MS, XL-MS increases the chance of identifying a protein-protein binding site, pinpoints the exact interacting residues, and contributes explicit distance restraints for complex modeling. Their integration allows for the generation of more precise, high-confidence models of protein interfaces (13, 40).

HDX-MS offers valuable information about protein dynamics and flexibility while standard biophysical techniques such as X-ray crystallography and cryo-EM provide static structures of a protein. As each approach brings forth unique information, combining HDX-MS with either X-ray crystallography or cryo-EM can complement one another and provides a more comprehensive picture of protein interactions. The distinctive ability of HDX-MS to capture conformational changes and dynamics was highlighted in a recent protein interaction study (41). Several promising sites for antibody neutralization on SARS-CoV-2 spike protein were detected by HDX-MS and would have been overlooked if investigators had relied only on a snapshot of the protein complex from X-ray crystallography.

Employing both HDX-MS and cryo-EM, a study of the interaction between transcription initiation factor σS and its activator protein Crl revealed an allosteric structural change at one site among all HDX-predicted binding sites (42). The allosteric site would have not been discovered based on the cryo-EM structure alone. Similarly, one of the HDX-predicted binding sites would have been a false positive based on the HDX-MS data alone. The combination of HDX-MS and cryo-EM unveils information that is unattainable by each individual technique. Besides distinguishing allostery, the benefits of this combined approach were thoroughly reviewed by Engen and Komives (43); examples of which include protein quality control, protein folding, and the process of large complex assembly and their mechanisms (43).

## HDX-MS Experimental Limitations Complicate Accurate Prediction of Residue-Residue Interactions

**Figure 2:**
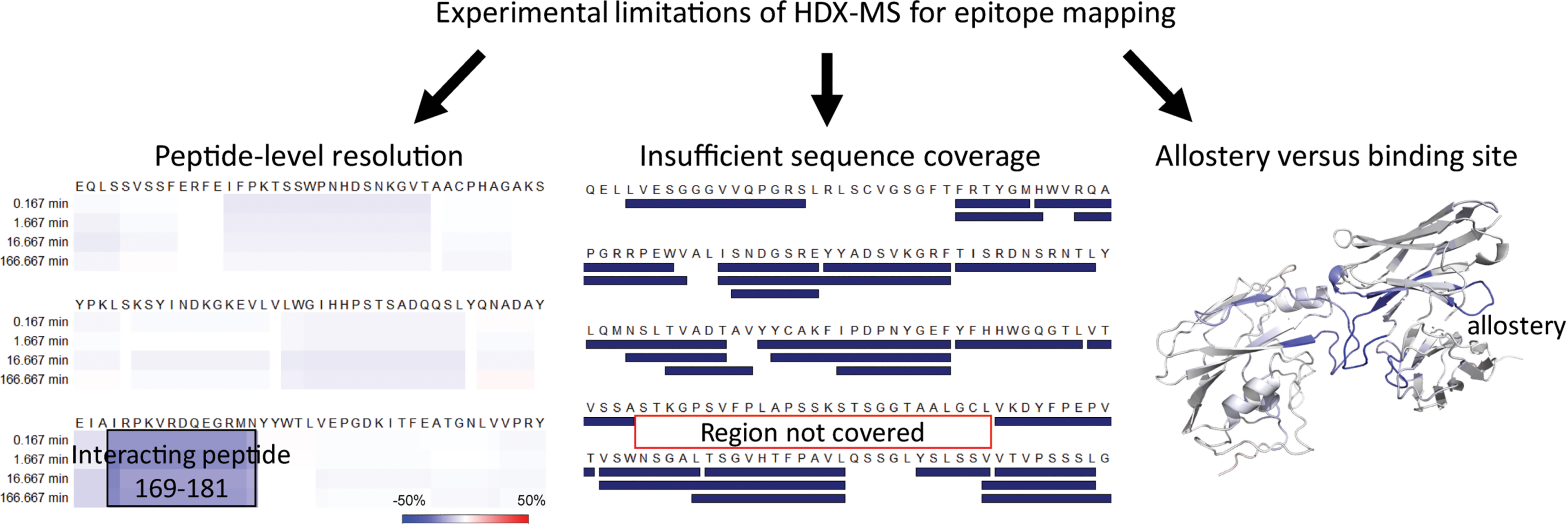
Experimental limitations when using HDX-MS for epitope mapping.

Emerging as a potent epitope mapping technique, HDX-MS comes, however, with several limitations. A brief introduction of the major inherent limitations of HDX-MS and some alternative or complementary approaches currently being used, along with their respective advantages and disadvantages, is presented below (see also **Figure 2** and **Table 1**).

**Table 1:** Current alternative or complementary experimental approaches and their pros and cons regarding each HDX-MS experimental limitation.

HDX-MS limitations	Alternative/complementary approaches	Short description	Advantages	Disadvantages
Peptide-level resolution	Gas-phase fragmentation: electron transfer dissociation (ETD) or electron capture dissociation (ECD) (24, 39, 44–47)	-Peptide ions are fragmented by ETD/ECD instead of CID. The c_k_ and z_k_ fragment ions report the nascent D content of the associated fraction of the parent peptide ion	-With gentle ESI conditions, fragments from ETD/ECD are accompanied by little to no H/D scrambling and can be solved to determine the D occupancy with resolution approaching residue level	-H/D scrambling can still occur without the optimized ion optics and gentle ESI conditions -Lack of easy-to-use and reliable software for ETD/ECD data processing -Impractical for routine implementation
Insufficient peptide coverage (specifically with large complexes, highly glycosylated Ag, and disulfide-bonded Ab)	Ion mobility spectrometry (IMS) (38, 45)	-Incorporation of IMS after the chromatography step deconvolutes unresolved overlapping peptides in chromatographic separation	-Increases the resolving power for overlapping mass-spectra and allows for identification of more peptides	-Challenge in routinely incorporating IMS in HDX-MS experiment (complicated experimental setup)
	Enzymatic deglycosylation of the glycoprotein (48–50)	-PNGase F prior to HDX-MS labeling	-Easy to implement -Reduces complexity of resulting peptides -Enhances detectability of glycosylated regions of the protein	-Risk destabilizing native structure of the Ag and can lead to aggregation -May misinterpret Ab-Ag interacting site
		-PNGase A or PNGase H+ after HDX-MS labeling	-Allows characterization of the native conformational dynamics and interaction at the glycosylation sites	-Requires offline pepsin digestion and manual sample injection into the LC-MS system
	Disulfide bond reduction for Ab (19, 38, 51)	-The chemical reductant TCEP is commonly added to the quench buffer at high concentration	-Protein becomes more protease susceptible and increases sequence coverage	-Can deteriorate both LC and MS performance
	Improve digestion efficiency (52–54)	-Multiple replicate pepsin digestions -Alter digestion conditions (e.g., off-line digestion, denaturants, etc) -Change to or supplement with another protease	-Produces more reproducible peptides -Generates many new short and overlapping peptide fragments due to different cleavage specificities of different digestive enzymes	-Material and time cost of experiment increases
Discern the difference between direct binding interface and allosteric conformational change	Complementary experiments and assays (10, 11, 13, 20, 25, 32, 55, 56)	-Site-directed mutagenesis followed by functional assays -Disulfide trapping in cells -Chemical crosslinking with MS -Kinetic millisecond HDX-MS (TRESI-HDX)	-Provides additional information to increase the certainty and better define the directly contacting regions	-Might need to try multiple approaches to reach a conclusion

### Peptide-Level Resolution

In most commonly used experimental setups, HDX-MS reaches peptide-level resolution, and individual residues’ contribution remains uncertain. When protonated peptide ions collide with neutral gas upon collision-induced dissociation (CID) - a typically-employed ion fragmentation method, CID increases the vibrational energy of the ions and leads to extensive intramolecular H/D scrambling (44). As a result, HDX-MS fails to achieve residue-specific resolution with CID employed and only provides the mass change for an entire peptide fragment (calculated as the centroid value difference of a peptide isotopic envelope at two time points) (24). There has not been a widely established MS method to locate single amino acid residues to which deuterium is incorporated (20, 45, 57).

An experimental solution to this challenge is the usage of electron-induced fragmentation methods such as electron transfer dissociation (ETD) and electron capture dissociation (ECD). In ETD/ECD with very gentle ion source conditions, D scrambling rarely happens because the internal energy deposited upon the recombination of an electron with the positive peptide ion remains localized, causing bond cleavage at the site where the electron is captured (11, 46). Nevertheless, gas-phase scrambling can still occur and needs to be diminished for different peptides through meticulous optimization of ion fragmentation and ESI parameters (24, 39). In principle, the ETD/ECD experiment enables single residue resolution through the calculation of mass differences between fragment ions (c and z ions) differing by exactly one overhanging amino acid. While different acid-stable proteases can be exploited to generate overlapping peptides, obtaining peptides that differ by only one residue and overhang through the entire protein sequence is unlikely. As ETD/ECD fragmentation is most efficient with peptides at high charge states, some peptides will be unamenable to this fragmenting method, preceding its application as a routine method for localize deuterium uptake at individual amide level (58). Overall, HDX-MS ETD/ECD fragmentation has promising potential to provide amino acid resolution but there is no guarantee that residue resolution, even only for the sites of interest, will be achieved (44, 45).

### Insufficient Sequence Coverage

HDX-MS experiments depend heavily on identifying reproducible peptides spanning the whole protein sequence. There is no threshold on what peptide coverage level is qualified as acceptable in the HDX-MS community (19). The goal is always to maximize the sequence coverage and to generate as many overlapping and unique peptides as possible to increase the sequence resolution in the optimization stage prior to HDX-labeling. To achieve this goal, the emphasis is on optimizing various HDX-MS conditions including quenching, digestion, and chromatography separation in the optimization stage (59). Insufficient peptide coverage or improper peptide identification in HDX-MS limits the ability to detect protein-protein interactions.

Large complexes contribute to this challenge in HDX-MS analysis because too many peptic peptides are produced. Given the restraints of the quench conditions in HDX, it is challenging to acquire chromatographic resolution for all the peptides generated. This results in overlapping mass spectra and complicates proper HDX-MS identification (38). In a recent report from Garcia et al., HDX-MS sequence coverage was significantly reduced when using a whole virus sample because of overlapping mass spectra from too many viral proteins present (60). One way to increase the resolution and reduce mass spectral overlap in HDX-MS experiments is to incorporate ion mobility spectrometry (IMS) after the chromatography step. Overlapping peptides in chromatographic separation can be separated in the gas phase with IMS, thus allowing the identification of more peptides in large complexes. However, more work is needed to routinely incorporate IMS in the overall HDX-MS experiment (45).

Highly glycosylated proteins, such as viral envelope proteins, pose a special challenge in HDX-MS experiment analysis. For glycoproteins, the substantial heterogeneity of the glycans combined with the broad specificity of pepsin digestion produces a diverse pool of peptic glycopeptides, leading to poor MS signal intensity of individual glycopeptides. As a result, severely reduced sequence coverage around the glycan sites is observed, significantly impacting the utility of HDX-MS to analyze such proteins (51). In Puchades et al., all residues immediately surrounding the hemagglutinin glycosylation sites lacked coverage (25). This challenge can be addressed by enzymatic deglycosylation of the glycoprotein with peptide-N4-(N-acetyl-β-d-glucosaminyl)asparagine amidase F (PNGase F) - the most common enzyme used prior to the HDX-MS labeling step (48). However, deglycosylation before labeling may alter the protein-protein interaction and prevent correct identification of antigenic sites, as some Abs carefully navigate around glycans. Deglycosylation also risks destabilizing the proteins and can cause aggregation. PNGase A and PNGase H+ are enzymes of choice when it is desirable to remove the N-linked glycosylations after labeling to preserve the native conformational dynamics and interaction of the glycoproteins (49, 50). Nevertheless, these enzymes require offline pepsin digestion and manual sample injection into the LC-MS system, which can be more labor-intensive than the automated sample injection system conventionally employed in the epitope mapping experiment. There are strategies to enrich the glycopeptides for MS analysis, but, to date, none have been developed for routine implementation in HDX-MS epitope mapping experiments (61, 62).

In addition to the highly glycosylated Ags, Abs with their disulfide-bonded regions add complexity to HDX-MS analysis. Peptides containing disulfide bonds are resistant to digestion, thus producing complicated fragment ion spectra and severely compromising interpretation. To reach an acceptable coverage of Ab sequences and facilitate HDX-MS analysis, a high concentration of TCEP is commonly added to the quench buffer to increase its disulfide reduction efficiency during the quench period (19, 38). On the other hand, too much TCEP can deteriorate LC and MS performance (51).

The stochastic behavior of pepsin contributes to difficulties in HDX-MS analysis. Although pepsin prefers to cleave between hydrophobic residues, it is still a non-specific protease with unpredictable cleaving pattern (63). Peptides generated from pepsin digestion display a great diversity of sequences; however, only highly reproducible and ubiquitous peptides can be used for further study and sequence-level comparison of deuterium content (52). Therefore, multiple replicates are needed to identify peptides reproducibly after pepsin digestion. When multiple replicate digestions under the same condition fail to result in reproducible peptides covering a region of interest, another strategy to increase coverage is to alter the digestion conditions by including additives such as denaturants or by using an activated pepsinogen coupled column. One other option is to change to or supplement with another enzyme (i.e., aspartic protease such as rice field eel pepsin) (53). A commercially available co-immobilized, dual protease column combines pepsin and type XIII protease from Aspergillus into a single packed column. The combination of their complementary specificities was shown to enhance the digestion efficiency of IgG molecules compared to pepsin or type XIII protease alone (54).

An acceptable sequence coverage percentage entirely depends on each practitioner’s own standard, their preliminary knowledge of the protein interaction, and the purpose of the project. Specifically in epitope mapping studies, the complementarity-determining regions (CDRs) of an Ab are often the regions of special interest. If the sequence coverage is perceived to be unsatisfactory or the regions of interest are not covered after optimization, the HDX study stage may be cancelled. It is not uncommon to encounter cases from time to time where HDX-MS does not provide any useful information on the binding sites of the proteins of interest. It is also important to note that even when the sequence coverage percentage is high, there is always a possibility of false negative results because a section of the protein sequence is not covered. Fortunately, this difficulty is one of several that computational docking can alleviate.

### Allosteric Effect Being Indistinguishable From the Binding Interface

Another limitation of HDX-MS is that protection due to protein-protein interaction can be confounded by allosteric effects. HDX-MS fails to discriminate between the direct binding sites and the remote conformational change resulted from complexation (11, 25, 32). Often complementary experiments are carried out to validate the direct binding sites such as site-directed mutagenesis (20). This approach can be laborious and expensive, with the occasional occurrence of false-positive results. Disulfide trapping in cells is another method that can verify the binding interface and validate MS data in physiologically-relevant conditions by introducing two cysteine residues, one per interaction partner, at selected positions within their potential interaction interface and examining the formation of disulfide bonds with oxidizing agents (13). The employment of XL-MS as a complementary approach can also distinctly detect conformational changes induced by protein aggregation from the reduction in solvent exposure (55). For modeling the complex, XL-MS contributes unambiguous distance restraints, unlike HDX-MS. The kinetic millisecond HDX-MS (TRESI-HDX) method utilizes the early (millisecond) labeling time where conformational equilibria are unsettled, thus allowing the HDX-MS signal to develop as the binding event occurs before the allosteric event (64). The millisecond deuterium labeling takes place in a capillary mixer incorporated into a microfluidic chip (65). The sample is then quenched, digested, and ionized rapidly. However, only epitope peptides from Ags can be identified. It is difficult to identify paratope peptides from the Ab *via* TRESI-HDX-MS because the pepsin-linked agarose resin used on the chip makes pepsin digestion less efficient, resulting in insufficient peptide coverage on the Ab. Furthermore, distinguishing binding events from allosteric events with TRESI-HDX-MS depends heavily on the time it takes for allostery to develop, which varies from case to case (32). Besides the above, many other methods can be used to validate directly contacting regions, such as low-resolution cryo-EM, immunodiffusion in gel, Ab competition, surface plasmon resonance, etc (56, 66).

## Computational Methods Try to Estimate the Residue-Specific Resolution of HDX-MS Experimental Data, Simulate HDX-MS Data From Protein Structure, and Explain Underlying Factors Contributing to the Observed HDX-MS Data

Computational methods are being used more frequently to tackle the inherent limitations of the HDX-MS technique. In this section, we focus on the state of computational methods in improving the resolution in HDX-MS experimental datasets and simulating HDX-MS data for native model selection through parallel comparison with the observed deuterium uptake profile. The complementarity of protein modeling and HDX-MS will greatly benefit from a detailed understanding of factors underlying the HDX mechanism.

### The Protection Factor (PF) Is a Biophysical Concept Used to Describe Hydrogen-Deuterium Exchange Behavior

**Figure 3:**
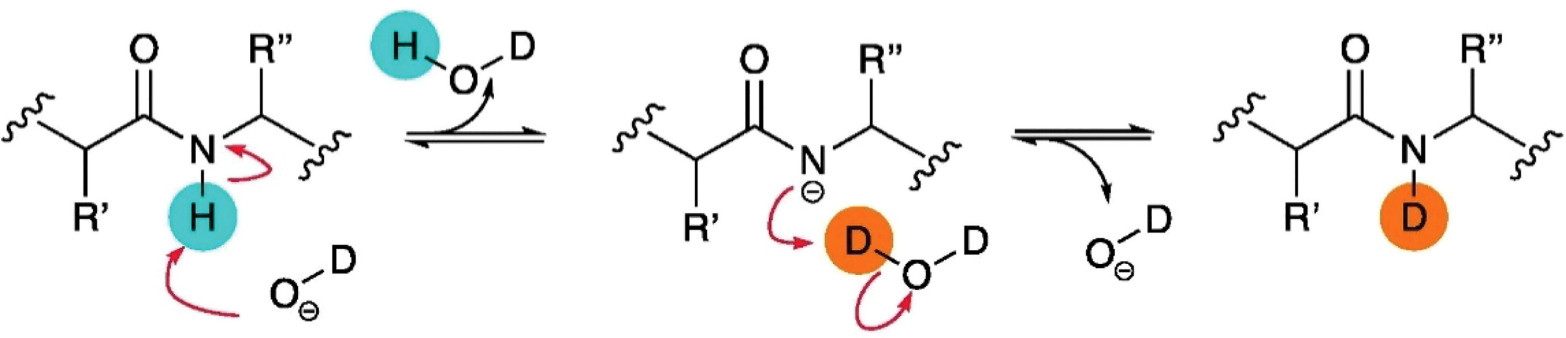
Schematic of base catalysis HDX of amide backbone protons in solution. Figure is reproduced from (57).

The H-D exchange reaction occurs at neutral pH in most experiments and predominantly adheres to a base catalysis mechanism. The amide proton is attacked by a deuterated hydroxide ion, leaving the amide nitrogen atom negatively charged (**Figure 3**). The amidated anion then removes a D+ ion from another D_2_O molecule to get re-protonated (57).

The key to investigating protein structure using HDX-MS is that the conversing rate from N-H to N-D is modulated by the protein structural attributes. The overall exchange mechanism can be described by the equilibrium below:



$$\text{N}-{\text{H}}_{\text{closed}}\overset{k_{\text{op}}}{\underset{k_{\text{cl}}}{⇄}}\;\text{N}-{\text{H}}_{\text{open}}\overset{k_{\text{ch}}}{\underset{{\text{D}}_{2}\text{O}}{\to }}\;\text{N}-\text{D}$$

*k*_op_ and *k*_cl_ represent the rate constants for the opening and closing reactions. *k*_ch_ is the “chemical” rate constant or the intrinsic and maximal exchange rate of N–H → N–D conversion measured for each residue amide N-H when they are solvent-exposed and without hydrogen bonds. The combination of these three rate constants is unique to each N-H group in a protein.

The N-H groups represent a continuum of hydrogen deuterium exchange mechanisms. If the rate of the opening/closing transition is lower than the chemical exchange rate (*k*_ch_ ≫ *k*_cl_), chemical exchange occurs quickly after conversion to the solvent-exposed form (i.e., the amide exchanges as soon as the very first opening event) and the observed rate (*k*_HDX_) is governed by the rate of structural opening (*k*_HDX_ = *k*_op_). This kinetic regime is called EX1. Under physiological conditions, EX2 is the dominant kinetic regime where the reconversion of the solvent-exposed to the protected state occurs much faster than the rate of chemical exchange: *k*_cl_ ≫ *k*_ch_ (17). Thereby, only a fraction of the available amide hydrogens will undergo deuterium exchange before resuming the closed conformation. The HDX exchange rate for EX2 regime is



$$k_{\text{HDX}}=k_{\text{ch}}\left(\frac{k_{\text{op}}}{k_{\text{cl}}}\right)$$

*k*_ch_ is dependent on the pH, temperature, and the protein primary sequence (i.e., the neighboring residues) and can be calculated from these parameters (67, 68). The only variable depending on the protein structure-related features is *k*_op_/*k*_cl_ ratio – the equilibrium constant of the closed-to-open state of N-H reaction (K_op_). The inverse of this equilibrium constant (K_op_^-1^) is the so-called protection factor (PF) (17, 29, 39).

The PF (*k*_ch_ */k*_HDX_) illustrates the degree of reduction in the observed exchange rate compared to the intrinsic exchange rate of a backbone amide hydrogen, in relation to the protein structure. Therefore, PF is a representation of how protein conformational properties hamper an amide from deuterium exchange. The concept of PF is especially important in folding simulations and can be used to infer local structural stability at specific sites on the protein (69, 70).

### Computational Approaches Enhancing the Resolution in HDX-MS Experimental Datasets Through the Estimation of PF Values or Equivalently Exchange Rates

Multiple mathematical and statistical methods have been developed to delineate exchange rates at the residue level of HDX-MS datasets with optimized fragmentation patterns (71–74). These predicting methods comprise a mathematical algorithm to assign potential HDX rates into equivalence classes for individual residues from overlapping peptide fragments (74), weighted residue-by-residue averaging of the HDX exchange midpoints (73), or a Bayesian modeling statistical method to assess the significance and magnitude of HDX differences between two states, which provides an estimate of ΔHDX for each residue given enough data available (72). Some studies localize the incorporated deuterium in peptides from the isotope distribution pattern. This approach computed trail deuterium uptake value for each amino acid in the protein sequence to achieve the best global goodness-of-fit to the experimental isotopic envelope of each observed peptide (71). This approach functions optimally with well-defined isotopic envelopes provided by high-resolution data. Improved versions of the method were reported (75, 76); however, the computational cost is quite expensive and thus restricts the method application to only small proteins weighing less than 30 kDa (76). Also, based on isotope envelope analysis, Hamuro et al. combined their isotope envelopes global-fitting algorithm with wide labeling time windows (eight orders of magnitude) and ETD to achieve residue-specific resolution (77, 78). Another computational method exploits overlapping peptide fragments from HDX-MS datasets cleaved by a mixture of pepsin and protease type XIII (79). A linear optimization iteration is performed to quantify the deuterium incorporation in segments from overlapping peptides, followed by a non-linear iteration to quantify exchange rates for all amides.

Overall, while the above computational methods demonstrate the viability of determining HDX rate at residue-resolution level, they are being adopted relatively slowly for conventional use. The reasons are due to the complex data processing procedures and the demanding experimental requirements. This is because most approaches, specifically those utilizing global fitting iteration, depend largely on high-quality data with an abundance of overlapping fragments, multiple time points, wide time windows, and resolved isotopic envelopes (79, 80). These requirements limit their use on most available HDX-MS datasets where the fragment patterns and redundant coverage are far from optimal. A statistical method developed by Skinner et al. was proposed to yield estimates or restraints of PFs for individual amides based on overlapping fragments regardless of the quality of input data (80). Nevertheless, the caveat is that the solution is ambiguous since the method can only provide an estimation of plausible distributions of the PFs (80).

### Computational Methods Predict HDX-MS Data (PF and Deuterium Uptake Level) for Comparison With HDX-MS Experimental Values to Improve Protein Model Selection

**Figure 4:**
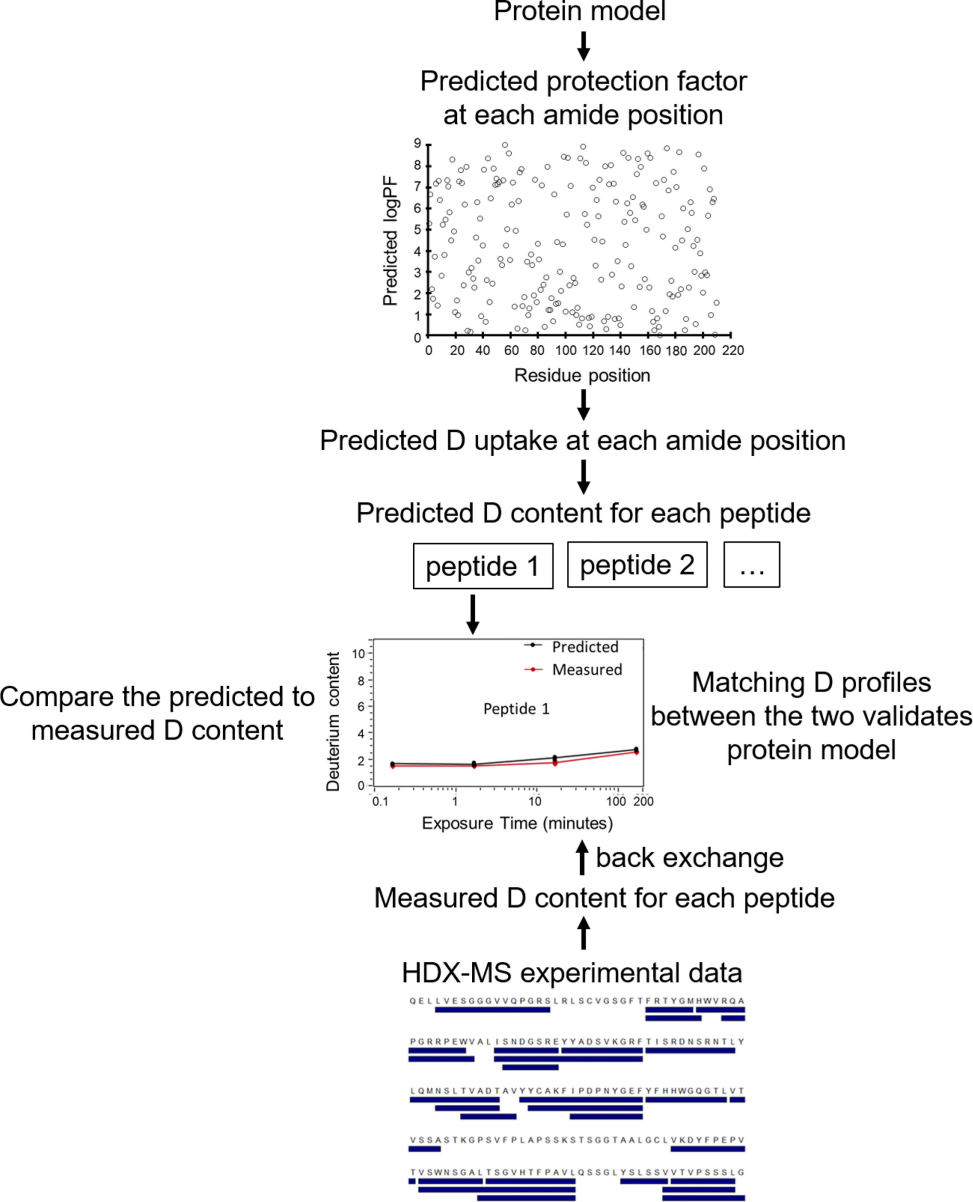
Flowchart of HDX-MS data simulation to validate plausible protein models. For each protein model, PF at each amide position is predicted. From PF and the intrinsic exchange rate *k*_ch_, the D value of each residue is calculated. HDX-MS data for each peptide (from the experimental peptide list) is generated by summing D uptake of the member residues. The simulated HDX-MS profiles of protein models are compared with the experimental data normalized for back exchange. Matching D profiles of multiple peptides validates the protein model, facilitating differentiation between native and non-native models.

Simulating HDX-MS result from protein model is a promising approach for protein structure prediction. By comparing experimental deuterium uptake with predicted deuterium profiles derived from protein models, native configurations can be validated (**Figure 4**). This approach requires the ability to accurately predict HDX-MS PFs from a protein structure, which has proven challenging. The details of this will be discussed in the next paragraph. The predicted PFs are used along with the intrinsic exchange rates *k*_ch_ to calculate *k*_HDX_ for each residue (Equation 2), from which their deuterium level is computed at user-defined labeling times. The deuterium uptake over contiguous residues of peptides present in the HDX-MS experimental sequence coverage list is integrated to estimate peptide isotope uptake data. Unique HDX-MS patterns are simulated for each protein model, facilitating model ranking and validation of native models through pairwise comparison to HDX-MS experiment data. Back exchange also needs to be accounted for. Deuterium loss from back exchange is easier to correct for during HDX-MS experimental data acquisition than through computational approximation, by measuring a fully deuterated control sample. Nevertheless, this extra step is disregarded in a majority of the reported HDX-MS datasets. While deuterium losses can be approximated computationally (68, 81), the varying experimental conditions such as temperatures, buffer solutions, pH, and LC eluent concentrations render these estimates inexact (82). Error in back exchange correction contributes to some of the disagreement between experimental and computationally predicted HDX-MS data (39, 82).

### Many Different Methods Have Been Developed to Predict PF From Protein Structures

Methods to estimate HDX-MS behavior of proteins through PF prediction have a unique combination of metrics. Each one incorporates different features of protein conformation to varying extents to derive PFs including solvent accessibility, hydrogen bond, electrostatic force, and solvation (39, 82–89). Simulation methods for PF estimation generally belong to one of the two groups: fractional population models and empirical models.

The first group - fractional population models, exemplified by the COREX method, exploits the connection of PF with the free energy difference between a protein folded and unfolded states (90). PF of a residue is derived as an equilibrium constant from a structural ensemble in an MD simulation and is computed by fractioning the total number of states where an amide is buried and “closed” to the total number of states where it is exposed and “open” (89).

The second group - empirical models, associates PF with protein structure-related features and uses structure-based scoring functions to approximate PF. The widely used phenomenological approximation method is an example of this group (83, 91). PF is characterized as the sum contributions of hydrogen bonding and amide burial (the number of heavy atoms surrounding the amide), with each term scaled by a previously defined weighting factor. Despite being underperforming, this method benefits from the ease of implementation with promising potential for high-throughput scoring of an ensemble of modeling outputs (92–94). The calculation of HDX data from a protein atomic models based on the phenomenological expression is now feasible for researchers using a software package called HDX ensemble reweighing (HDXer) developed by Bradshaw et al. (93). In a recent study, this technique was applied to simulate the deuterium uptake of the top docking poses to compare with the experimental data (36). The simulated HDX profiles of the two docking complexes aligned well with the empirical data, further validating the selected models, and highlighting the advances being made in the field of HDX prediction.

Also belong to this group of empirical models, a recent method called protection factor prediction based on protein motions (POPPeT) incorporated additional information of secondary structure features and protein motions (95). The POPPeT method was shown to predict amide hydrogen PFs more accurately than both the phenomenological approximation and the COREX method. However, POPPeT was based on information about HDX-enabling protein motion, which is rarely available and is thus not considered a full-fledged PF prediction method by its authors (95).

Moreover, high accuracy in amide hydrogen PF prediction do not necessarily translate into high accuracy in deuterium uptake at peptide level (95). A recent study compared the accuracy of HDX-MS simulation from nine different PF predicting models (96). Using the same set of proteins, each model was evaluated by how well it matches HDX-MS experiment result. Mohammadiarani et al. concluded that the fractional population models outperformed conventional empirical models. Nevertheless, even the most reliable models in this study still had large errors (around 40%) and low correlation coefficients when predicting experimental HDX-MS data.

Overall, attempts to reliably estimate deuteration profiles that match closely with the experimentally determined HDX-MS data based on predicted PFs have been proven challenging so far. In addition, PF formulations that require usage of MD simulations or other sampling methods are computationally expensive and thus, are impractical for high-throughput ranking of docking simulation models. Indeed, further testing on the capacity of these methods to identify native conformations among others is severely lacking.

### Simulated HDX-MS Patterns From Protein Decoys Can Benefit Structure Prediction

The Borysik research group is the only group, to date, to have extended to work on simulating HDX-MS in structure prediction and docking (92, 94). Using PF predicted from the easy-to-implement phenomenological approximation method, Borysik and his colleagues predicted HDX-MS outputs for two monomeric proteins (barnase and alpha-lactalbumin) and two homomeric proteins (enolase and serum amyloid P), each with 1,000 models and compared them with experimental HDX-MS data (94). HDX-MS simulation generated for crystal structures of two homo oligomeric proteins correlate significantly less with the experimental data than the monomeric proteins. This might be because the algorithm used to predict PF was originally trained on monomeric globular proteins only and was never optimized for usage on large multiple chain proteins. Indeed, when this phenomenological approximation method was applied to help select native conformations among the docking outputs in another study, optimization was required to tune the PF expression for protein interfaces (92). PF of proteins obtained in their bound configurations was redistributed towards those values simulated for the unbound states, akin to incomplete binding where a fraction of each protein would populate unbound conformations in the mixed samples. In both studies, pairwise comparisons of HDX-MS simulated output with experimental data was shown to be sufficient to discern native structures from non-native ones, as well as selecting the native docking pose (iRMSD<0.7 Å) without the necessity of further data analyzing or manual interpretation (92, 94). It was demonstrated that the predicted PFs, even when poorly determined, can still be adequate for simulation of HDX-MS data or HDX-MS difference data (produced by subtracting the simulated isotope uptake of the bound from the unbound states) that effectively evaluate and discriminate between poses. As the two studies were performed to only a few proteins, future work is in need to fully assess the potential of simulated HDX-MS data in high-throughput structure evaluation. Nevertheless, these studies revealed the promising power of HDX-MS in protein modeling and how this area would greatly benefit from improved prediction of PFs from protein atomic structures.

### Factors Contributing to PF Are Incompletely Understood

Progress on improved simulation methods is pending upon a deeper understanding of HDX structure determinants. Factors commonly thought to contribute to amide backbone protection, making the overall exchange rate constant *k*_HDX_ smaller than the intrinsic exchange rate *k*_ch_ are intramolecular hydrogen bonding and limited solvent accessibility (17, 97). A study by McAllister and Konermann reexamined this notion by exploring the correlation between experiment and simulation (98). In their 1-μs all-atom simulation of ubiquitin, only 57 out of 72 amide exchanges were accounted for through hydrogen bonding or solvent exposure. Some amide hydrogens were solvent accessible and did not participate in hydrogen bonding but were found to be protected from exchange. Electrostatic factors and hydration could potentially be contributing factors for these observed discrepancies between the simulation and HDX pattern. The ambiguous understanding of the HDX-MS process has led to the apprehension that HDX events are too intricate to be expressed in a formula (99). More study on the correlation between theoretical mechanisms of HDX-MS and its experimental measurement is necessary to improve future models.

## HDX-MS Experimental Results on Ab-Ag Interaction Have Been Combined With Computational Docking

Computational docking is a common method to predict Ab-Ag interaction when a complex crystal structure is unavailable and has been combined with HDX-MS to better map epitopes (100, 101). In this section, we will focus on different practices for implementing HDX-MS in Ab-Ag docking as restraints in sampling and model generation and as filters in model selection.

### Overview of Computational Ab-Ag Docking

The protein-protein docking process involves two main steps: effectively sampling various docking configurations and accurately ranking the decoys by free energy score. There are many algorithms for protein–protein docking such as RosettaDock (102–104), DOT (105), HADDOCK (106), ZDOCK (107), ClusPro (108), PatchDock/SymmDock (109), and FTDOCK (110). In this paper, RosettaDock in the Rosetta software suite is chosen to exemplify a typical docking protocol (111).

RosettaDock is a Monte Carlo-based docking algorithm that employs rigid-body docking of two interacting partners and optimizes their side-chain conformation (102–104). The algorithm requires a structure of both proteins in an initial docking pose as input. This is either manually arranged if prior structural information about probable regions of interaction is known or is randomized through a global docking step (102, 112). RosettaDock starts with a low-resolution (coarse grain) docking step. A Monte Carlo search is performed with rigid-body movements (namely adaptive rotation and translational moves) around the surface of the binding partners being represented in centroid-mode. For Ag–Ab interactions, the search space for Ab is limited to the six CDR loops as they are known to be the binding sites for the Ag epitope (113). The best scoring model from the low-resolution stage is adopted for high-resolution docking (all-atom refinement). All-atom side chains of docking partners are restored in place of centroid atoms, followed by additional docking position refinement and side-chain optimization steps (112). The performance of a docking attempt and evaluation of the best model can be assessed by the total energy and the energetic enhancement of the interface (binding interface energy), as well as the Cα RMSD to the best scoring model.

Predicting a protein-protein interaction is challenging due to many possible docked conformations. Although Ab recognition of Ag is largely limited to the CDR loops, Ab-Ag interactions present a unique challenge for computational docking. The structural flexibility of the CDR loops, especially HCDR3, and the homology model-based inaccuracy of Ab can confound docking (114, 115). As conformational changes after complexation and error in Abs homology modeling are taken into consideration, Ab-specific moves have been incorporated into the RosettaDock protocol (SnugDock): refining of HCDR2 and HCDR3 loops after the low-resolution stage and sampling of V_H_–V_L_ orientations during the high-resolution stage (113, 116). Nevertheless, if motions upon Ag binding is substantial or the Ab models are substandard, it would be challenging, if not improbable to accurately predict the native binding structures.

**Figure 5:**
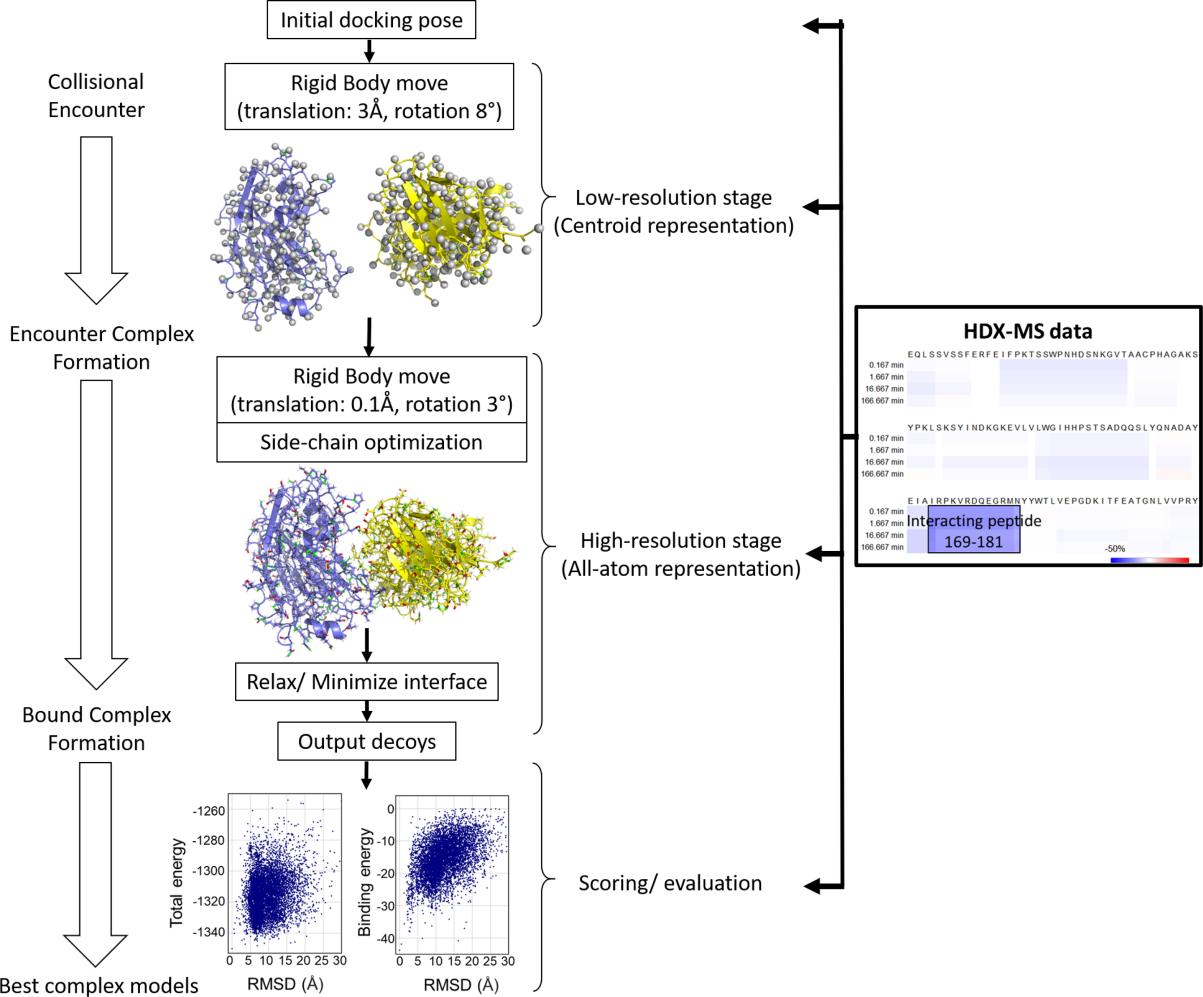
The protein-protein docking procedure from RosettaDock – a multiscale Monte-Carlo based algorithm with different stages for HDX-MS data to be incorporated. HDX-MS data can be applied to the sampling stage (including the arrangement of the starting pose, low-resolution docking, and high-resolution docking) as well as the scoring stage. Figure is adapted from (102).

The chance of achieving an accurate docking model will be enhanced by including experimentally obtained restraints - a strategy that has become increasingly popular (117, 118). Various experimental methods have been employed in combination with computational docking such as NMR, site-directed mutagenesis, electron paramagnetic resonance, low-resolution cryo-EM, and XL-MS (41, 117, 119–122). Here, we focus on how HDX-MS and computational docking are combined to identify the epitope of various Ab-Ag interactions. Given a diverse collection of restraints and filters in Rosetta, knowledge derived from HDX-MS experiments can be incorporated into the sampling strategy (**Figure 5**). For example, the starting docking pose is manually arranged to a configuration compatible with the experimental knowledge, which allows for the exploration of only a small region of conformational space and improves sampling density around the putative binding sites. Additionally, distance-based filters (or restraints) can be set during either low- or high-resolution docking stage or both to bias sampling towards docking poses compatible with the experimental data. Moreover, HDX-MS data can be applied to select native models, either manually or as filters or ideally being incorporated into the scoring function (**Figure 5**).

### Different Practices for Implementing HDX-MS as Restraints in Computational Ab-Ag Docking

In a recent benchmark study, blind Ab-Ag docking using current docking algorithms was shown to achieve a success rate of approximately 66% for near-native prediction using CAPRI (Critical Assessment of Predicted Interactions) criteria of Medium accuracy (115). Although there has not been any benchmark study to examine the success rate of HDX-MS in conjunction with Ab-Ag docking, evidence in the literature has pointed to significant improvement in docking performance with HDX-MS.

**Figure 6:**
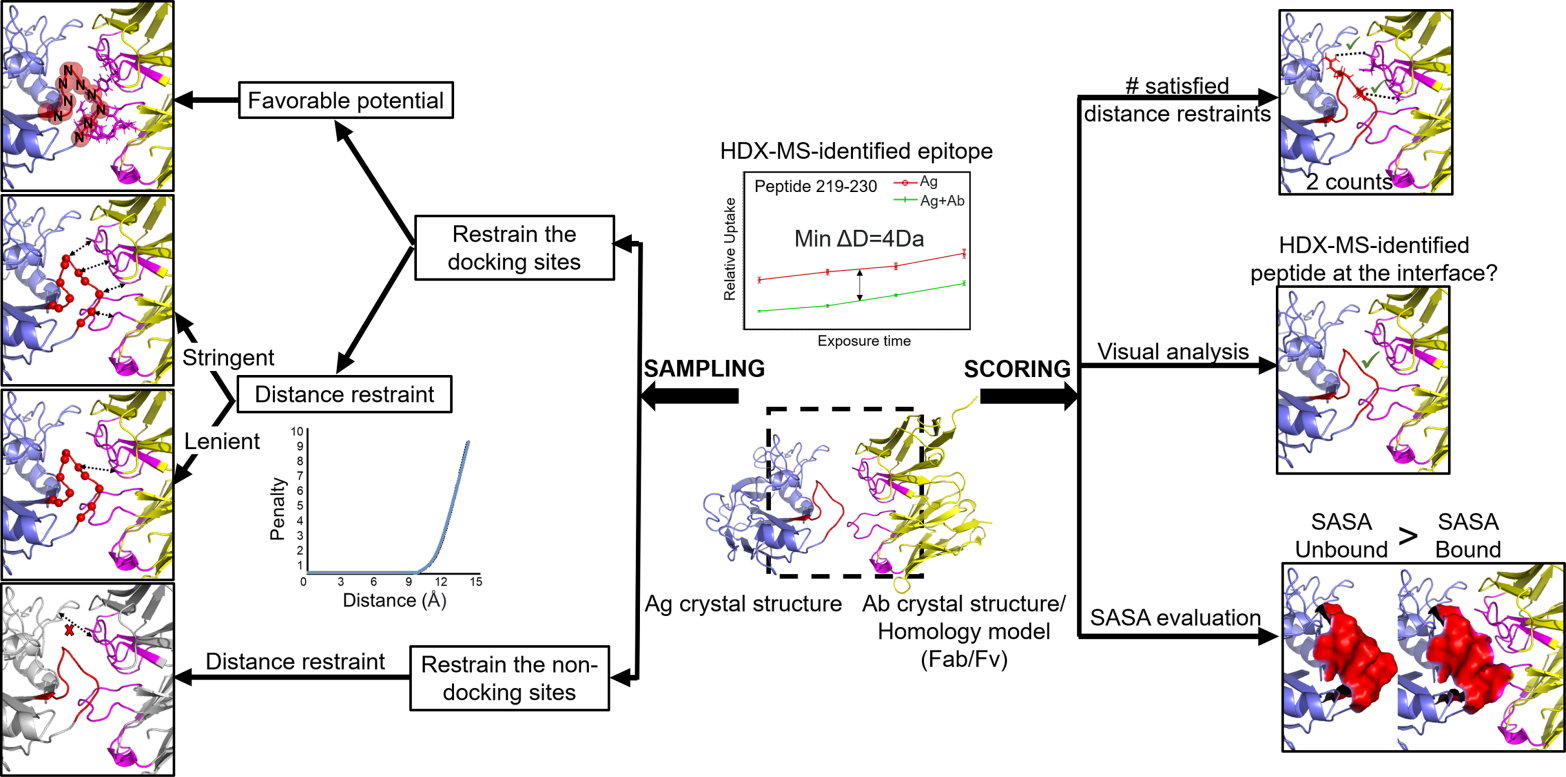
Different practices have been used to incorporate HDX-MS data into Ab-Ag docking. HDX-MS data are provided in form of identified epitope peptides (e.g., peptide 219-230 with a decrease in deuterium level of at least 4Da across 4 timepoints). Docking components: Ag crystal structure (blue; HDX-identified peptide: red) and Ab (Fab or Fv) crystal structure or homology model - (yellow; CDRs: pink). Sampling stage: **(A)** Restrain the docking sites. Favorable potential (red sphere) - spheres surrounding the backbone N atoms in the epitope are filled with favorable values. Each atom of the Ab lying within this sphere would contribute a favorable potential to the energy function. Distance restraint (left right arrows) - a minimum number of residues from each epitope peptide must be within a specified threshold distance to the Ab. Lenient approach: one residue from the epitope peptide to be at the interface. Stringent approach: the minimum number of contact residues correlates with the decrease in deuterium level (i.e., 4 residues in this case). **(B)** Restrain the non-docking sites: non-epitope peptides and non-CDR regions (grey) are blocked from docking using distance restraint. Scoring stage: HDX-MS data is applied as filters in form of: the number of satisfied distance restraints is - the higher the count (favorable contact) is, the more optimal a docked pose is considered to be; visual analysis - manually inspection of the docked poses on whether the epitope is at the interface; SASA evaluation - SASA calculation of the epitope peptide in the unbound form must be larger than the bound form.

HDX-MS signals have been combined with docking simulations in different protein modeling suites (i.e., MOE, PatchDock, ZDOCK, RosettaDock (102, 103, 112, 123) to derive Ab-Ag complex structures in several epitope mapping studies (e.g.,^11^, ^14^, ^27^, ^32^, ^36^). A summary of their protocols and docking performance is presented in **Table 2**. The ideal starting components for docking are crystal structures of unbound Ag and unbound Ab (in the form of Fab fragments or variable fragment Fv). In the absence of an experimentally determined Ab structure, a homology modeled Ab is often used instead, representing a more common Ab-Ag docking case. One study used models of HDX-predicted Ab peptides (32). The number of sampling poses ranges from 50,000 to 100,000. The detailed practices of implementing HDX-MS as restraints in computational docking vary between studies and are depicted in **Figure 6**.

**Table 2:** A summary of studies integrating HDX-MS and docking to predict Ab-Ag native poses and their performance.

Studies	Docking algorithms	Docked Ag	Docked Ab	Sampling and applying HDX restraints	Model selection	Evaluation
(27)	ZDOCK (in combination with ZRANK and RDOCK)	crystal structure	crystal structure (Fab) or homology modeled antibody (Fv) using RosettaAntibody protocol	-54000 poses were sampled in ZDOCK -All residues in non-CDR regions (Ab) and in non-epitope peptides (Ag) were blocked during ZDOCK, allowing the pairwise shape complementarity (PSC) scoring function of ZDOCK to penalize docked poses when the blocked residues are in the interface -The initial-stage docking poses were scored and ranked using ZRANK -The top 50 rigid-body docking poses were refined using RDOCK	-The top 10 poses from RDOCK refinement process were selected for evaluation -The RMSD value of the interface Cα atoms (iRMSD) was calculated by superimposing a docked pose onto the relax co-crystallized antigen-antibody complex. Docking poses with an iRMSD less than or equal to 2.5 Å are considered near-native structures or “hits” with the interface defined as all residues with at least one atom within 10 Å of the binding partner	-Compared to stand-alone docking, the HDX-MS-derived restraints significantly improved the docking results for one of the three testing Ab-Ag complexes: the number of “hit” poses among top 10 poses generated increased from four to seven, with the iRMSD of the highest-ranking pose being 1.4 Å to the complex crystal structure -Incorporation of HDX-MS data produced more tightly clustered docking poses for all three complexes and did not interfere with the result when the stand-alone docking already did well by itself
(32)	MOE	crystal structure	models of HDX-predicted antibody peptides were generated with MOE	-HDX-predicted epitope peptides were set as the docking sites	-Five (MOE) to ten (PatchDock and ZDOCK) molecular dynamics-minimized docking poses were selected for evaluation -Optimal poses are those with the highest numbers of energetically favorable contacts (“hit”) between the paratope peptides and the antigen, where a “hit” is regarded when a residue from a predicted epitope peptide located within 4.5 Å of a residue from the antibody peptide	-For all three software packages, computational docking with HDX-MS data produced more “hit” residues than docking without HDX-MS data. In other words, more ‘hit’ residues were detected for docking at the HDX-specified site compared to randomly selected sites -The crystal structure of the Ab-Ag complex is not available. Thus, it cannot be determined how much the iRMSD to the native structure improved with the incorporation of HDX-MS
	PatchDock			-HDX-predicted epitope peptides were set either as the docking sites or as volume-constraint pharmacophores		
	ZDOCK			-HDX non-epitope residues of the antigen were blocked as a scoring penalty		
(11)	MOE	crystal structure	Homology modeled antibody (Fab) using Bioluminate protocol v1.9 and MAESTRO v10.2	-100,000 starting poses were sampled using MOE -CDR restraints were applied by using an energy penalty to require that all poses contain a minimum number of residue contacts between HDX-predicted paratope peptides and these regions -The poses were further refined and scored using a full-atom potential (AMBER)	-The top 200 poses were evaluated for surface complementarity based upon AMBER complementarity score (24) and visual inspection of surfaces as implemented in the protein_contact_surfaces script implemented in MOE	-The best docking poses were proposed to be the Ab-Ag interaction model. The HDX-predicted peptides in this model were at the interface and were corroborated by the SASA analysis -No blind docking was done for parallel comparison
(14)	Rosetta	crystal structure	crystal structure (Fab)	-Restrict docking in Rosetta to HDX-predicted epitope of the antigen and the CDRs region of antibody -The docking poses were filtered by overall energy, binding energy, and satisfaction to HDX constraints -The best 500 models by binding energy underwent the protocol again	-An ensemble of 25 best-scoring models (by binding energy) that fulfilled HDX constraints were selected	-The best docking poses were proposed to be the Ab-Ag interaction model. Functional assays were performed, and the results endorsed the binding modes of the docked complexes -No blind docking was done for parallel comparison
(36)	PatchDock	crystal structure	Homology modeled Ab (Fv) using ABodyBuilder Fv prediction	-HDX-predicted epitopes were set as docking sites by adding a scoring parameter to PatchDock -The clustering RMSD was set at 4 Å	-The top 100 poses were evaluated for CDR inclusion at the interface and agreement to the alanine scan data. Among these, the top two poses were selected	- HDX profile simulation was performed using the ‘calc-HDX’ function of the HDXer tool for the top two docked structures -Comparison between the simulated ΔHDX versus the experimental ΔHDX further validated these poses, displaying a RMSD of deuterium exchange of 0.981 Å and 0.684 Å -No blind docking was done for parallel comparison

Not all of these studies were carried out in comparison to blind docking. However, those that did demonstrated a higher number of energetically favorable contacts in the docking poses and better interface root-mean-square deviation (i-RMSD) of the top models (27, 32). Aside from Ab-Ag complexes, docking studies with HDX-MS integration were performed for other protein-protein complexes (13, 40, 125). The incorporation of HDX-MS as restraints and filters was shown to enrich the native-like conformations in the prediction and accurately select high-quality, biological-relevant models that would not be the case for docking poses ranked by the regular binding scores.

### HDX-MS Data Are Applied as Restraints in the Sampling and Model Generation

HDX-MS difference identifies peptides at the protein binding interfaces. This information can be utilized as restraints during the generation of docked complexes to better focus the conformational sampling space. The restraints are generally in the form of distance restraints (score penalty) or favorable potentials. They facilitate one of the two goals: favoring the presence of residues in HDX-predicted epitope peptides at the interface or hindering residues outside of these peptides from participating in binding.

To propel the presence of HDX-MS predicted epitope peptides at the interface, these peptides are assigned as favorable docking sites (11, 14, 32, 36). This is accomplished by applying fixed distance restraints between residues in HDX-detected epitope peptides and residues in the Ab. Since most Abs interact with Ags through the CDRs, the restraints for Ab are often applied to residues in the Ab CDRs or residues in HDX-MS-detected paratope peptides if an HDX-MS experiment on paratope mapping was done. The interpretation of the distance restraint is that the sampling poses are required to contain a minimum number of residues from each epitope peptide within a specified threshold distance to certain residues in the Ab, or an energy penalty is applied.

The distance restraint comprises three elements whose parameters are seldom explicitly stated. The first element is the minimum number of residues from each HDX-MS peptide to be restrained at the interface. The qualitative, lenient approach does not take into account the ΔHDX signal intensity. Regardless of the magnitude of the relative deuterium uptake difference in a peptide, the lenient, qualitative restraint only requires at least one residue in each interacting peptide to be at the interface (14, 125). A more stringent approach is to correlate this number with the deuterium uptake difference between the unbound and the bound states (125). Roberts et al. reported that one approach could offer more optimal results than the other depending on the protein it is applied to. While the stringent approach allows for quantitative HDX-MS data interpretation in form of restraints, it runs the risk of overestimating the number of residues in the HDX-MS peptides that truly contact the other molecule, which would bias the docking process to incorrect configurations. This is because the increased level of solvent protection is not only caused by protein interaction but also allosteric effects, especially in the case of structural stabilization. Therefore, the interpretation of HDX-MS interacting peptides must be confident for the stringent approach, perhaps by additionally inspecting the 3D structure of the unbound protein. Otherwise, it might be better to interpret HDX-MS data as lenient, qualitative restraints. Perhaps a hybrid approach can be used where the stringent restraints are applied to confidently identified HDX-MS peptides (either through visual inspection of 3D structure or complementary data from other experiments) and lenient restraints are applied to the remaining HDX-MS peptides. We have not seen such a hybrid approach reported.

The second element is the nature of the distance restraint. Unlike chemical crosslinking, HDX-MS fails to provide a numeric distance threshold between specific atoms for docking. Thus, for implementing HDX-MS restraints, the distance and the atom of each of the two residues for which this distance being measured are improvised by researchers. They vary between studies, such as 10Å between Cα atoms of two residues (14) or 7Å between the backbone nitrogen atom of one residue and any heavy atom of another residue (125). One study examined a range of distance values and suggested that the optimal distance value can be slightly different for different protein systems (125). The third element to consider is the magnitude of the applied energy penalty function. A too harsh score penalty will likely hamper the sampling process by trapping structures in a local energetic minimum, which might result in a uniform population of complex models with the wrong conformation.

Another way to favor HDX-MS peptides as docking sites, besides using distance restraints, is through added potentials, although this is less commonly used. The docking study of Roberts et al. was performed with DOT rigid-body docking calculation (105, 125). In one protein, backbone nitrogen atoms of HDX-MS predicted peptides were assigned favorable potential. Each atom in the other protein residing within specific distance cut-off to these nitrogen atoms would contribute a favorable potential to the free energy score. A variety of sphere sizes and values within them were examined and the optimal values were chosen (125). While docking with added favorable potentials endeavors to maximize the number of contacts between specific residues of one protein to another, it neglects that one molecule must simultaneously contact all the interacting peptides on the other molecule. Meanwhile, the distance restraint has greater flexibility to enforce simultaneous contact by multiple regions.

Instead of requiring HDX-MS peptides to be buried at the interface, a different approach to apply HDX-MS data to the sampling stage is excluding regions with identical deuterium exchange profiles before and after complexation from the interface (20). In this approach, all residues in non-epitope peptides and non-CDR peptides are blocked from docking using distance restraints (27, 32). This approach relies on the notion that a common HDX-identified peptide is larger than the actual contacting segment and thereby contains most of the true contacting residues (27). As a result, it is safe to assume that residues in non-epitope peptides fail to participate in protein-protein interactions. However, this assumption is inapplicable to regions rich in proline because these are the “blind spots” in HDX-MS experiment; no inference can be drawn about them from HDX-MS data.

### HDX-MS Data Are Applied as Restraints in the Scoring and Model Selection

A major challenge in protein docking is identifying native models among the entire ensemble of docking outputs. In studies leveraging HDX-MS data to evaluate docked models, the bulk of the evaluation relies on the total score and binding energy computed by the docking software (13, 14, 32, 40, 125–127). Based on these energy functions, an initial list of 50,000-100,000 models can be curated to an ensemble of the top 25-2,000 models. HDX-MS data are then incorporated to distinguish correct complexes from these top-scoring models. One crude way to utilize HDX-MS data for model selection is visual inspection (14, 40, 126, 127). The top 10-25 models are manually inspected for agreement with HDX-MS data: the binding regions with reduced exchange rate upon complexation should reside in the interface. In contrast, regions with constant exchange rate should be absent from the interacting regions. Visual analysis is commonly performed in studies combining docking with both XL-MS and HDX-MS where XL-MS is used to provide distance restraints during sampling and HDX-MS is used to narrow down the best complex models at the final stage (40, 126, 127).

Other studies implemented HDX-MS data as filters to eliminate incorrect configurations. The filter can be based on solvent accessible area (SASA) calculations where SASA of the interacting regions predicted by HDX-MS in the bound protein is compared with the SASA of its unbound form. A model is considered to be accurate when the SASA of these regions in the docked complex are all smaller than that of the unbound (13). Another form of HDX-MS filter is the number of distance restraints being satisfied. Docking models are eliminated if they fail to fulfill a minimum number of distance restraints (125). Using this criterion, Deng et al. ranked the docking models where the optimal poses had the highest numbers of favorable contacts between the two proteins (32). It is worth noting that applying HDX-MS data as distance restraints during both the sampling and the scoring stage can result in incorrect configurations due to overfitting. To avoid overfitting models when using HDX-MS data, some HDX-MS restraints should be omitted from the sampling stage and instead used to validate the ensemble following refinement. Nevertheless, this approach only works if there are multiple HDX-identified interacting peptides, which might seldom be the case for many HDX-MS experiments.

Overall, although many studies have been conducted using different software packages, there is currently no common best practice to implement HDX-MS restraints or guidance on how to use these restraints in docking simulations, nor has a consecutive method been developed for how to select models fulfilling HDX-MS restraints.

## Current Docking Simulations Have Yet to Capture the Complexity of HDX-MS Data

**Figure 7:**
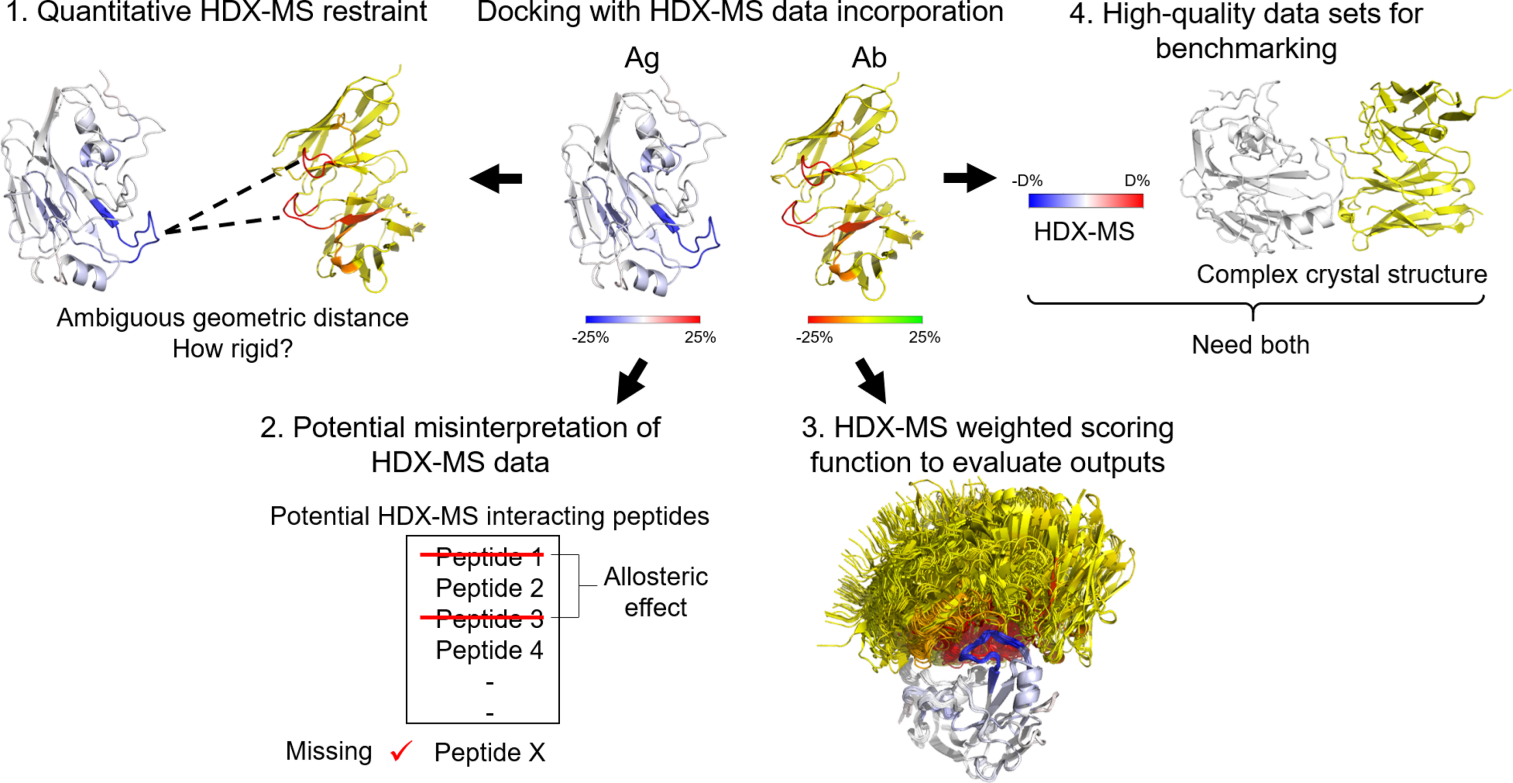
Challenges of HDX-MS data incorporation into docking. 1) Quantitative HDX-MS restraint: HDX-MS data is incapable of providing quantitative geometric distances between atoms, thus, hindering its application to the sampling process. 2) Potential misinterpretation of HDX-MS data. True positive signals can be undetected due to insufficient coverage and inherent blind spots or overlooked. False positive signals are very probable due to manual data interpretation, noise, and allosteric effects. 3) HDX-MS weighted scoring function. The evaluation of docking poses based on HDX-MS data is still largely qualitative. 4) High-quality dataset for benchmarking. HDX-MS data and crystal structure of an Ab-Ag complex in most cases are exclusively available. There is a need for establishing a database for HDX-MS epitope mapping data.

Although hybrid methods for combining experimental data and docking simulations have been quite well-developed for techniques such as cryo-EM, NMR, XL-MS, or electron paramagnetic resonance (117), an optimized method for integrating HDX-MS data into computational docking, in general, and for Ab-Ag complexes, specifically, is needed. A brief overview of the challenges associated with this task is presented in **Figure 7**. The opportunities that these deficiencies provide are described in the following paragraphs.

### Quantitative HDX-MS Restraint

As mentioned above, relatively crude atom-atom restraints have been most commonly used to restrain the distance between residues in HDX-predicted interacting peptides, with no regard to the intensity of HDX-MS signals. The geometric distances between specific atoms deduced from HDX-MS data are ambiguous for usage as quantitative restraints in model building. There is an opportunity for benchmarking a set of optimized parameters constituting HDX-MS distance restraints. Ideally, the rigidity of the restraint correlates with the magnitude of the relative deuterium uptake difference for each peptide to some degree (a balance between the stringent approach and the lenient approach).

While the number of native-like structures can be significantly enriched in the prediction with HDX-MS data being applied as restraints, the expectation of accurate docking with HDX-MS where near-native configurations are always generated is unreasonable. Regardless of how HDX-MS data are applied to restrain the docking sites (either as distance restraints or favorable added potential), the inherent properties of HDX-MS data mentioned above might mislead docking experiments (125). First, HDX-MS are sparse, in which information is insufficient to fully constrain the structure. Second, HDX-MS provides no information about proline residues. Both properties can move the best ranking complexes away from the correct conformation.

### Potential Misinterpretation of HDX-MS Data

HDX-MS data can be ambiguous. True positive interactions can go undetected due to insufficient coverage and inherent blind spots (peptides rich in proline) in HDX-MS experiments. Even when detected, a true interacting peptide can be overlooked in HDX-MS experiments. Such a case was demonstrated by Pandit et al. by comparing the interactions of three different Ab-Ag complexes between HDX-MS data and X-ray crystallography data (27). Although 35 contacting residues were detected by X-ray crystallography, only 20 of them were correctly identified by HDX-MS. There were nine contact residues undetected (in the blind spot regions) and six contact residues detected but their deuterium uptake remained unchanged upon complexation (27). Why this happens is poorly understood. As described above, the process of HDX-MS of proteins is highly complex and translation between HDX-MS and structure is still being investigated (98, 128).

In addition, HDX-MS data can be non-specific with moderately high false-positive signals. There are recommended thresholds on the difference in D levels that would statistically be considered as significant (129). However, due to different experimental set-ups and auxiliary information known to the researchers, manual review and assessment are often called for and various studies assign the statistically significant differences in HDX-MS peptides differently. Thus, it is challenging to properly account for experimental errors and noise in HDX-MS experiments. False positives are also possible because of allosteric effects, as mentioned above.

Overall, HDX-MS data are sparse and maybe partially incorrect or incomplete. As a result, a promising approach applying HDX-MS as experimental restraints should balance the weighting of HDX-MS data in qualitative and quantitative docking for improvement in docking accuracy.

### HDX-MS Weighted Scoring Function

Evaluating docked models by HDX-MS has been a qualitative procedure. A better way to quantify the agreement of docking models with HDX-MS data would be to alter the scoring function for each calculated metric to consider the sparseness of the HDX-MS data. HDX-NMR data has been incorporated into a scoring function used for computational *de novo* protein structure prediction (130). Considering residual solvent exposure and structural flexibility, the weighted scoring function improved RMSD of the selected model in 12 out of 15 proteins and by 1.42 Å on average (130–132). This study paves the way for future development of a scoring algorithm for HDX-MS sparse data in docking protocols, which would significantly enhance Ab-Ag complex model selection.

### High-Quality Datasets for Benchmarking

One challenge for developing a computational method that incorporates sparse experimental HDX-MS data in Ab-Ag docking is high-quality datasets for computational benchmarking are insufficient. Ideal Ab-Ag complexes for benchmarking will require both HDX-MS datasets and crystal structures of the Ab-Ag complex. Nevertheless, most Ab-Ag complexes with HDX-MS data lack crystal structures because HDX-MS experiments are often performed when the proteins are unamenable to X-ray crystallography. This deficiency provides the opportunity for establishing a database to deposit HDX-MS epitope mapping datasets. The development of an HDX-NMR incorporated scoring system was made possible thanks to a large HDX-NMR dataset on protein folding and stability available *via* the Start2Fold database (130, 133). Similar to Start2Fold, a framework for deposition and analysis of HDX-MS experimental data targeting protein complexes would tremendously benefit integrative computational modeling effort (19, 51).

Suppose the obstacles mentioned above could be resolved for computational modeling, a typical HDX-MS experiment will be sufficient to restrict the sampling space and output an ensemble of near-native, high-resolution Ab-Ag complex models. HDX-MS could be used more reliably and with greater throughput for epitope identification for Ab engineering.

## Future Directions

HDX-MS is uniquely suited to characterize protein interactions, even for complexes that are challenging to study by other biophysical techniques. The biggest intrinsic limitation of HDX-MS experiment is its resolution being at the peptide-level and being limited by the observed sequence coverage. New developments over the past decades have continuously pushed the limits of what is feasible for HDX-MS (51, 57, 134, 135). Improved separation and digestion techniques like ion mobility and combined proteases are being used to obtain different sets of overlapping peptides, and thus increase the resolution through a subtractive approach. With the continued development of mathematical algorithms estimating single amide deuterium uptake from HDX-MS datasets and the increase in accessibility to gas-phase fragmentation methods along with the advancement in their data analysis software, residue-resolution HDX-MS may become more established and routine in the future.

Advances in HDX-MS prediction models have been made and were applied to discriminate between conformational poses by a comparison to experimental HDX profiles. Nevertheless, accurate computational simulation of HDX-MS data from protein sequences has proven challenging so far as the combination of all structural determinants underlying HDX kinetics are not fully understood. The development of successful methods relies heavily on the greater availability of residue-level experimental HDX data for proteins with known structures (136). This deficiency is being addressed by the establishment of the Stat2Fold database and the emerging protocols in HDX-MS experiments enabling single residue HDX datasets. Collaboration between experimental and computational researchers will provide more usable data for model development to be collected, which provides more structural insights on HDX-MS and increases the prospect of accurate HDX prediction models. In another approach to improve protein complex modeling, the incorporation of HDX-MS into molecular docking, HDX-MS data is often applied qualitatively using distance restraints to corroborate the produced docking models, with little regard to the intensity of the experimental signals. Enhanced sampling and ranking of docking models will require benchmarking of more sophisticated methods that exploit deeper layers of HDX-MS data. The future of this area is moving towards docking protocols that enable quantitative usage of HDX-MS data as restraint and weighted scoring functions to accurately generate atomic-level models of Ab-Ag complexes. Overall, an integrated platform of HDX-MS and protein complex modeling, once established, will be a powerful tool in elucidating Ab-Ag binding interfaces with high-resolution and minimum turnaround time, especially in the absence of other structural information. This advancement will propel HDX-MS to become an indispensable tool for reliable, routine, and high-throughput epitope mapping in the near future.

## Author Contributions

The manuscript was mainly written by MT and CS with contributions of all authors. All authors have given approval to the final version of the manuscript.

## Funding

This work was supported by a grant from the National Institute of Health (NIH) (R01 GM073151) and the RosettaCommons. Work in the Meiler laboratory is supported by the NIH (U01 AI150739 and U19 AI117905).

## Conflict of Interest

The authors declare that the research was conducted in the absence of any commercial or financial relationships that could be construed as a potential conflict of interest.

## Publisher’s Note

All claims expressed in this article are solely those of the authors and do not necessarily represent those of their affiliated organizations, or those of the publisher, the editors and the reviewers. Any product that may be evaluated in this article, or claim that may be made by its manufacturer, is not guaranteed or endorsed by the publisher.
